# Alternative Mating Tactics in Male Chameleons (*Chamaeleo chamaeleon*) Are Evident in Both Long-Term Body Color and Short-Term Courtship Pattern

**DOI:** 10.1371/journal.pone.0159032

**Published:** 2016-07-13

**Authors:** Tammy Keren-Rotem, Noga Levy, Lior Wolf, Amos Bouskila, Eli Geffen

**Affiliations:** 1 Department of Zoology, Tel Aviv University, Tel Aviv 69978, Israel; 2 Blavatnik School of Computer Science, Tel Aviv University, Tel Aviv 69978, Israel; 3 Department of Life Sciences and the Mitrani Department of Desert Ecology at the Blaustein Institutes for Desert Research, Ben Gurion University, Beer-Sheba 84105, Israel; University of Regina, CANADA

## Abstract

Alternative mating tactics in males of various taxa are associated with body color, body size, and social status. Chameleons are known for their ability to change body color following immediate environmental or social stimuli. In this study, we examined whether the differential appearance of male common chameleon during the breeding season is indeed an expression of alternative mating tactics. We documented body color of males and used computer vision techniques to classify images of individuals into discrete color patterns associated with seasons, individual characteristics, and social contexts. Our findings revealed no differences in body color and color patterns among males during the non-breeding season. However, during the breeding season males appeared in several color displays, which reflected body size, social status, and behavioral patterns. Furthermore, smaller and younger males resembled the appearance of small females. Consequently, we suggest that long-term color change in males during the breeding season reflects male alternative mating tactics. Upon encounter with a receptive female, males rapidly alter their appearance to that of a specific brief courtship display, which reflects their social status. The females, however, copulated indiscriminately in respect to male color patterns. Thus, we suggest that the differential color patterns displayed by males during the breeding season are largely aimed at inter-male signaling.

## Introduction

Alternative mating tactics among males have been extensively studied and documented in various taxa, such as insects [1] ‏ isopods [2], mites [3], fish [4–7], amphibians [8], and reptiles [9–12]. Several studies in vertebrates have shown that the link between body size and mating tactics is often reflected in male rivalry over territories [13–14]. In these cases, larger males, which have a physical advantage over smaller ones, maintain the mating tactics of territorial males. In contrast, smaller and younger males, with little fighting experience, may find fighting costly and risky [15]. Thus, smaller males may select the "sneaker" tactic that, by avoiding territorial behavior, reduces conflict with dominant males [16–19].

Alternative mating tactics can be expressed in body color polymorphism, in which each tactic is characterized by its own distinctive color pattern [12, 16, 20–24]. In some of these species, small males use body color to disguise themselves from larger males by similarity in appearance to females [25–27]. Through appearance similarity, smaller males may consequently reduce aggression by the dominant males and gain access to the females guarded by these dominant males (e.g. giant cuttlefish [28] and lizards [12, 26–27]). The fitness of these different tactics is not equal [13], and Maynard Smith [17] has suggested that mating tactics in males are maintained by a frequency-dependent selection.

Alternative mating tactics have also been found in the Chamaeleonidae. Cuadrado [29–32] reported two distinctive mating behaviors in the common chameleon (*Chamaeleo chamaeleon*), in which the larger dominant males used mate-guarding behavior, while the smaller subordinate males did not guard females or defend territories, but acted as floating breeders that actively searched for females. Cuadrado [29–31] also reported intrasexual body color polymorphism, which is expressed seasonally. The typical body color of the common chameleon in Spain is green for both males and females throughout the year, except during the breeding season when only some males and some females remain green while others alter their body color to brown [29–31]. However, Cuadrado did not link between mating tactics, body size, and seasonal body color changes.

Following Cuadrado's results [29–32], we formulated two hypotheses. First, we hypothesized that alternative mating tactics in male chameleons are linked to body color and size. We predicted that larger males will win male-male encounters, and that these winning males will have a color pattern significantly different than of losers (i.e. subordinate males). Furthermore, we predicted that winning males would behave differently in the presence of receptive females (e.g. mate guard) relative to the losers (e.g. sneakers). We have tested this hypothesis by a series of trials where we observed the behavior of male-male and male-female pairs of various sizes and color patterns. Second, we hypothesized that females prefer to mate with dominant males. We predicted that females would prefer to mate with males displaying the courtship color patterns associated with dominance. We have tested this hypothesis through observations on female sexual preferences in a series of male-female encounters.

## Materials and Methods

### Ethics Statement

The common chameleon is a protected animal under Israeli law. Collecting them from the wild for our behavioral trials required a permit from the Israel Nature and Parks Authority, which we were granted annually (permit no. 31153/2008, 32296/2009, 37394/2010, 38014/2011, 38579/2012). This study complies with all Israel regulations on ethical treatment of wild animals under scientific investigation.

### Study animals

The common chameleon occupies park forests and plantations in the Mediterranean region of Israel [33] and southern Spain [34]. While their adjustable camouflage allows them to approach prey and avoid predation, their coloration may also be dependent on other factors such as temperature, social interactions, and breeding state [31]. In Israel, chameleons are active mostly during the warm months (May–November). Mating occurs in July–September, and during October–November females deposit 14–47 eggs, which remain underground for 10 months of incubation [35].

Hatching occurs in late August. Hatchlings are brownish during their first few weeks of life and perch on dry brownish grass. During November these juveniles start perching higher on evergreen bushes and small trees, and a partially green pattern appears on their skin. The fully green appearance in juveniles is only observed in December, which implies an age of 3–4 months [36]. One-year old males are small and brown during their first breeding season, and are already sexually mature and sexually active (TKR, personal observation). During the breeding season, all juveniles in the wild are at the age of a few weeks. This age cohort is sexually immature and spends their time at ground level [36]), thus, not interacting with adults.

### Study site and data collection

We conducted the study along the Maharal creek on the Mediterranean coast, at the foothills of Mt. Carmel in Israel (32°38' N, 34°58' E). The study site is a relatively dry habitat covered by Mediterranean forest. In summer, mean maximum and minimum daily temperatures are 30 and 21°C, respectively, and relative humidity averages about 70%. Mean annual rainfall is 550 mm, and falls only during the winter months (November–March; [36]). Fieldwork was carried out during May–December in 2008–2012. We also studied the timing of courtship, copulation, and egg depositing in the field (unpublished data) and used this information to define the annual cycle in our chameleon population. We defined May-August as "before the breeding season", September-October as "the breeding season" and November-December as "after the breeding season".

We collected chameleons from vegetation using a spotlight during the night when they were asleep and their bodies are light in color and reflective. A total of 277 sexually mature adult individuals (186 males and 91 females) were used in this study. Within this sample size, we sampled different individuals for each set of trials. To minimize stress, the chameleons were kept for up to 24 hours (often less than 12 hours) in separate 35 X 20 cm terraria, all placed in a shady area and inside a screen cage to prevent predation. Keeping them outdoors allowed the animals to experience the same air temperature and humidity conditions as in their natural environment. We did not provide food or water because the animals were held during the nighttime, when they are not active, and released several hours past sunrise when the daily trials were done. Therefore, we assumed that they did not require either.

All the individuals were weighed and sexed according to the presence of developed male hemipenial organs, a swelling at the cloaca which was easily detect by eye from May to December. Individuals were used in a single trial session to prevent pseudo replications and carry-over effects, and released back at the capture site the following day. We individually marked all captures prior to release by clipping the tip of 1–3 nails using a fingernail cutter. This marking procedure took only a few seconds and the hand-held animals showed little resistance to it. The clipped nails regrew a rougher tip, which served to identify recaptures but did not affect the animals' ability to climb branches [30]. We conducted 15–20 surveys annually to collect the animals. During these surveys we also recorded the location of captures, and recapture data was used for confirmation of seasonal and annual shifts in body color.

### Recording body colors and color patterns

We documented body colors and patterns during the morning hours. We placed each of the 109 individuals (38 individuals collected before the breeding season and 71 during the breeding season) separately on a 2 m long measuring stick, which was located horizontally 1 m above the ground. The stick had smooth polycarbonate rolls affixed to each end, which prevented the animals from descending. Each individual was placed in turn on the middle of the stick and its color and pattern on both sides of the body were documented by a series of digital images. The animals often walked naturally along the stick, allowing us to record color patterns (S1 Fig). In this position, the body is fully extended and the legs are away from the lateral sides. We cropped each image such that only the full extent of the body was left for further color pattern analysis.

We used a Canon EOS D30 digital camera and macro lens Canon 100-400mm f/4.5–5.6L IS USM, which saves the data as RAW formatted files, to record colors in color images, and patterns and contrast in grayscale images. The camera was placed on a tripod 2 m from the focal animals. Photos were taken under natural sunlight, without a flash. Each photo included a color standard in the form of a white ruler running along the horizontal stick. We standardized image colors with a study-specific 'white standard' (e.g. [37]) using the spectral reflectance of the white spaces in the ruler along the stick, and Photoshop software (Adobe Systems Software). Our approach resembles that of Bergman & Beehner [38], where photos taken in the field were calibrated using the GretagMacBeth ColorChecker chart. We provided a photo of our white ruler and a GretagMacBeth ColorChecker chart for future calibration of our raw photos to any desired standard (S2 Fig).

The images were also used to measure the SVL of animals while on the stick in a fully extended position (e.g. S1 Fig). SVL measurements taken from handheld individuals using calipers or a measuring tape are not precise since the animals are highly flexible and tend to curl up when handled. We confirmed the accuracy of our measuring method in a preliminary study using three independent images of each of 10 individuals (data not shown). The mean coefficient of variation (CV) in SVL within individuals was 0.8%.

### Classification of color patterns

**Fig 1:**
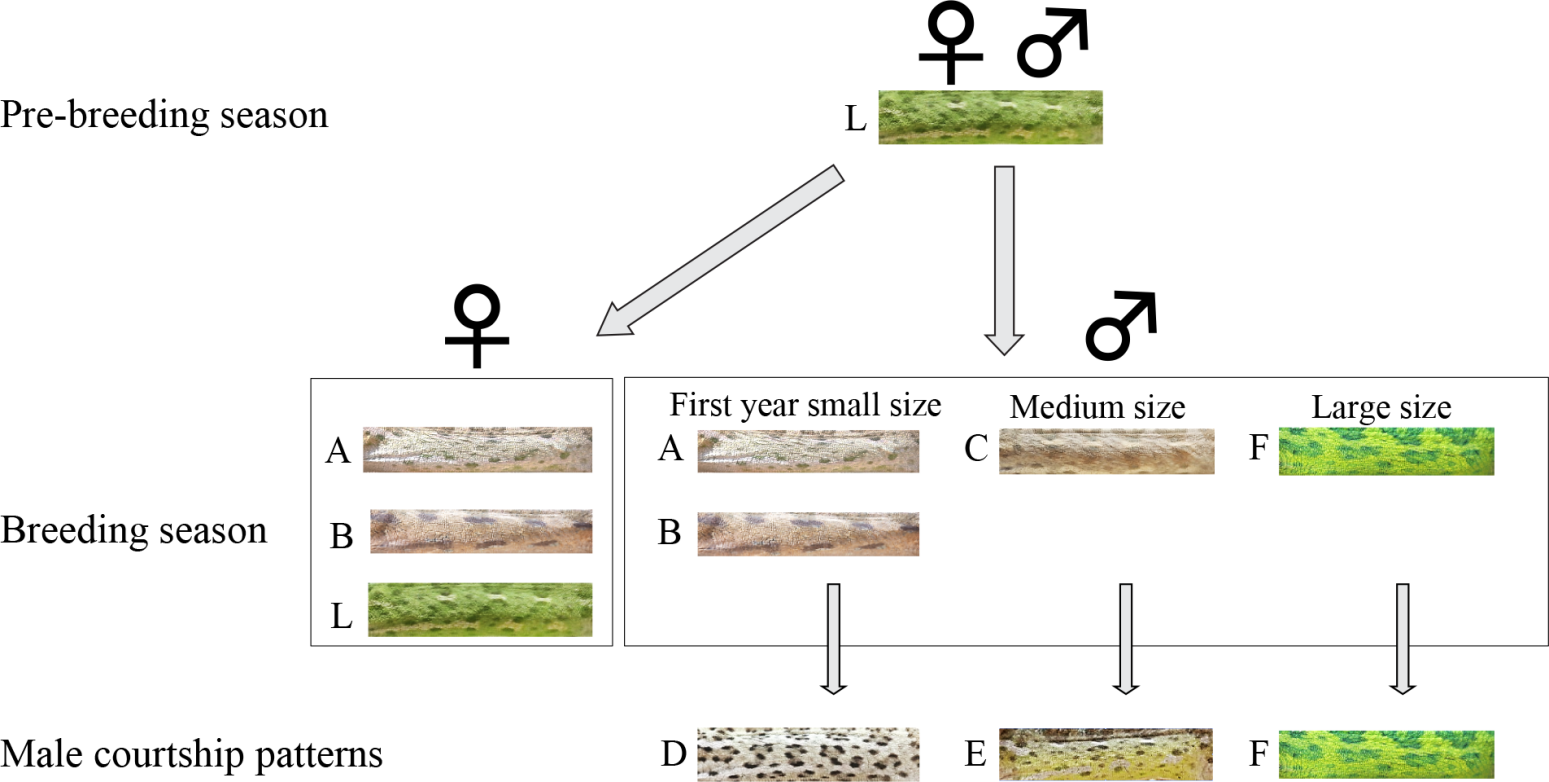
Flow chart showing temporal changes in body color pattern of common chameleons in Mt. Carmel coast. During the pre-mating seasons, animals have a unisex green pattern (L). During the breeding season, some females and all small-sized males changed to the brown A or B patterns, medium-sized males changed to the brown C pattern, and large males change to new green pattern–pattern F. During courtship, small-sized males used the D pattern, medium-sized males used the E pattern, and large-sized males stayed unchanged in the F pattern. These short-term courtship patterns corresponded to the long-term body color patterns (A, B, C, and F) observed during the breeding season.

Our aim was to associate between color patterns and social status. First, we documented color patterns of 109 individuals using images taken during our various trials (see below). Second, TKR classified the images into color pattern categories, as followed: (1) all images were split into subgroups by season, sex, and context (i.e., lone, and sex combination encounters). (2) in each subgroup images were visually classified to different pattern categories according to similarity in colors, stripes, and patches. (Fig 1).

To corroborate our manual classification of images into color patterns, we employed computer vision techniques, which were combined with statistical tests in order to validate the inter-pattern distinguishability. We represented the input image (7.5 Mb, 3504 X 2336 pixels) as a vector of thousands of measurements (i.e. a visual signature [39–40]). Each individual measurement contains very little information, but collectively the visual signature captures key aspects of the image's visual appearance. The structure of this system is based on insights derived from biological vision systems by including elements such as hierarchical processing, non-linear spatial pooling, and opponent color channel processing [39–40]. Note that although the parameters of the processing were based on the primate visual system, we do not know yet whether chameleons have color vision, although they have the potential for tetrachromatic color vision along with other diurnal lizards and birds [41]. In order to support reproducibility, the raw individual images used in the analysis are deposited in Dryad (doi:10.5061/dryad.9kd74), and the cropped images and the method codes are posted publicly on https://github.com/nogaor/Chameleon_classification. The implementation relies heavily on the single opponent descriptor of [40], using the authors' code and the default parameters. This encoding is an extension of the C2 gray image descriptor [39]. The C2 descriptor is calculated by computing the maximal similarities to a set of 1000 templates pooled across all image locations. In other words, what is recorded, per image, is the maximal similarity between each of 1000 templates and the best matching image location. The templates are simply selected, as a preliminary step, by sampling 1000 random image locations, taken from all images. In order to support patterns of different sizes, the 1000 templates are divided equally among four sizes.

Each image and each template is represented, for the purpose of the comparison between an image location and a template, by the C1 representation, which contains the output of Gabor filters at 16 different scales [39]. The Gabor filters serve as bar detectors, of the sort that is found in the primary visual cortex (V1) of mammals.

The modified C1 layer, used in the single-opponent descriptor [40], is augmented by an eight center surround channels. These channels correspond to cells in which the central receptive field is sensitive to light in one color, while the off-center receptive field is sensitive to another. The first two such channels are the red-green channels where a surround green regions is being subtracted from a central red region, or vice-versa. The other opponent pairs are yellow-blue, the less conventional red-cyan, and the luminance based white-black. The responses of the 8 resulting channels are rectified, by taking only the positive part, i.e., applying the absolute value operator. This biologically motivated step is done in order to maintain a positive firing rate.

Due to the inclusion of the color-opponent channels, the system distinguished images based on both color and spatial pattern. The fact that the representation of [40] is an extension of [39] for color images allows us to isolate the color component. While the latter is invariant to color (only affected by luminance), the former adds biologically-motivated color sensitive channels. In other words, by employing the baseline C1/C2 representation of [39] we are able to observe the distinguishability of spatial patterns independently of color information, and by employing the single opponent descriptor [40] we can observe the added value of color.

Our system distinguished images based on both color and spatial pattern. We tested distinguishability among patterns by using both color and grayscale images in the analysis. We used grayscale images to determine whether body patterns were constructed only upon color. We measured distinguishability between visual patterns based on statistical classification using direct measurement of distances between visual patterns. We used the Support Vector Machine (SVM, [42]) using the LibSVM software package [43], which is a popular binary classification algorithm, and measured the leave-one-out classification error (i.e., cross-validation). We used linear SVM with a constant parameter C = 1 and the number of support vectors varied between 50 and 70. To measure the distinguishability of two patterns, X and Y, we set aside one example from either class, and used the rest as the training set. In this approach the visual signatures of the training set are used in order to train the SVM classifier. This classifier is employed on the single test example and the predicted label is compared to the true label ('X' or 'Y'). The process is repeated multiple times, where at each turn a different photo is taken as the test example. The average classification success rate is recorded. For two very different classes we would expect this rate to be high, and vice versa. The P value of the classification success rate is computed using permutation tests. In each one of 10,000 randomizations, the labels are randomly permuted among all samples, and the distribution of success rates under the null hypothesis that labels and visual appearances are not linked, is estimated.

### Trial set-up

To test our hypothesis regarding the association between male body color, mass, and social status, we carried out a series of lone male and male-male encounter trials. We conducted all trials in an arena comprised of a 2.5 m high *Ficus benjamina* tree planted in a pot (29 cm high and 35 cm in diameter), and a measuring tape vertically aligned with the tree. This trial set-up allowed individuals free movement in all directions along the tree and thus they could easily move away from their opponent. In a series of encounter trials between two individuals, we defined dominants and subordinates by observing perch height, the tendency to descend off the tree, outcome of agonistic interactions (i.e. chases and fights), level of body inflation, and change in body color and pattern. All lone male trials and male-male encounters were recorded using a high-resolution camera as outlined above.

All lone male and male-male encounter trials were conducted during the breeding season in the morning hours under natural light and ambient temperature. For each lone male trial, we used a sexually mature male (n = 38), which was randomly placed on a tree branch and its behavior recorded for 20 min at 5 min intervals. For each male-male encounter trial, we placed two males (n = 23 pairs) on the same tree branch, with the first male placed one minute before the other to allow it to climb up into the foliage. Both males were chosen randomly in respect to body size, age, and color. Having two males in close proximity can result in immediate aggressive interaction. In the few cases of aggressive eruption (n = 3), when males chose directly to bite one another rather than chase or retreat, the observer ceased the aggressive escalation by removing one of the males. Bites left flat marks on the attacked animal but did not penetrate the skin. In such cases we did not define social status of the two opponents because it was not clear which was the winner.

Finally, we used the same trial set-up and conditions to test female preference for males. We hypothesized that females would prefer to mate with dominant over subordinate males. Trials were set during the breeding season. In each trial, we introduced a single male to each female, and each such pair participated in only a single trial (n = 42 trials). For each trial, the female was placed first on the tree trunk, and one minute later we placed nearby a randomly chosen male. During these trials, we recorded courtship behavior, the number of copulations and of rejections of males by females. Overall, we used 12 green, large-size and 30 brown, small-medium-size males in our trials. A total of 12 females were used such that each female attended a mean of three trials with 3 different males. Yet, each female was not attending these trials one after the other but was returned to its cage for a break of minimum of 20 min between trials. Females that showed a non-receptive color pattern during trials were excluded from the analysis.

We used logistic regression to associate body color (dependent variable) with other continuous independent variables (e.g. month and SVL). This type of regression fits nominal Y responses to a linear model of X terms using a logistic function, and is most suitable for a two-level response. Model fit was evaluated using the goodness-of-fit test (i.e. comparison between the full and the reduced models). Likelihood ratio test (χ^2^) was used to test the significance of each effect in the model. Random effects were added to the logistic model by using the generalized estimating equation (GEE) procedure. We used a contingency table to associate between two nominal responses (i.e. color and status or copulation and status). All calculations were carried out in JMP (ver. 10, SAS Institute Inc.) and SPSS (ver. 22, IBM). All the data used in the above analysis is provided in S1 Table.

## Results

### Classification of color patterns

Color patterns of adult individuals were documented as snapshot images. A database of 109 adult body images (a single image for each individual; 36 and 73 females and males, respectively) was visually classified a priori into six different color patterns (Fig 1), according to body color, distinctive ornaments, and contextual information (season, sex, breeding or non-breeding season, and social status).

**Fig 2:**
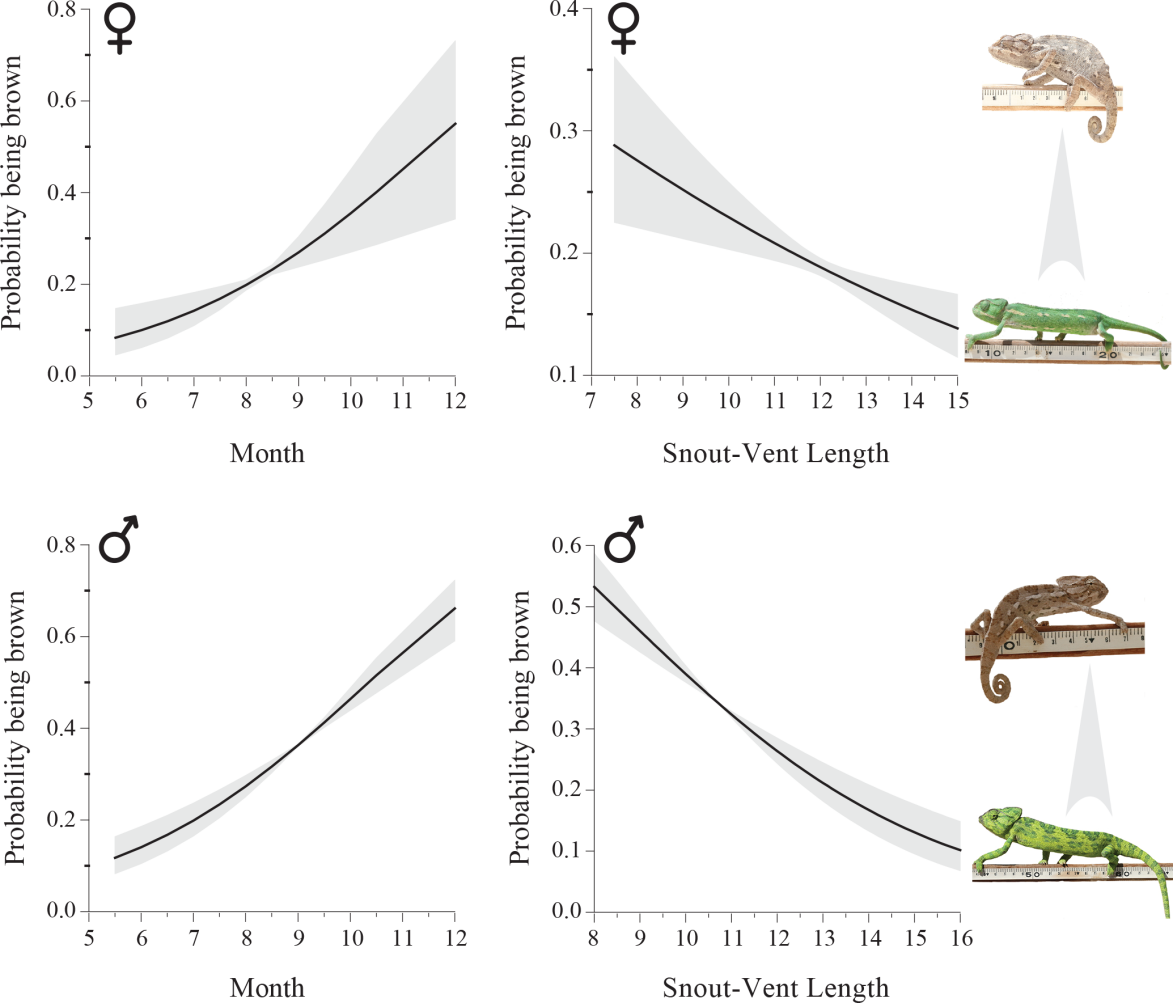
Predicted probability of being brown (± 95% confidence intervals in grey) as a function of month of the year and snout-vent length (SVL) in males (bottom) and females (top). The overall tendency to turn brown increased towards the breeding season (males; logistic regression, β = 0.416, likelihood ratio χ^2^_1_ = 57.1, P<0.0001, n = 148; females: β = 0.400, LR χ^2^_1_ = 11.6, P = 0.001, n = 91) and decreased with body size (males: β = -0.289, LR χ^2^_1_ = 47.5, P<0.0001; females: β = -0.123, LR χ^2^_1_ = 11.2, P = 0.001). The breeding season is during September-October.

Adult individuals were mostly green in color throughout the year. However, we observed a seasonal shift in body color of adult individuals during the breeding season. From June onwards, some of them were brown (displaying color patterns A or B or C; Fig 1), a trend that increased toward the peak of the breeding season (i.e., September; Fig 2). This trend was significant for both males (logistic regression, β = 0.416, likelihood ratio χ^2^_1_ = 57.1, P<0.0001, n = 148; Fig 2) and females (β = 0.400, LR χ^2^_1_ = 11.6, P = 0.001, n = 91). Body color also correlated with body length; larger individuals tended to be green and smaller ones were mostly brown (males: β = -0.289, LR χ^2^_1_ = 47.5, P<0.0001; females: β = -0.123, LR χ^2^_1_ = 11.2, P = 0.001; Fig 2). Year was set as a random effect in the above analyses. Our recapture data showed that all males exhibit brown body color during their first breeding season (n = 67), while some of them appeared in green color during later breeding seasons (n = 3, S3 Fig).

**Fig 3:**
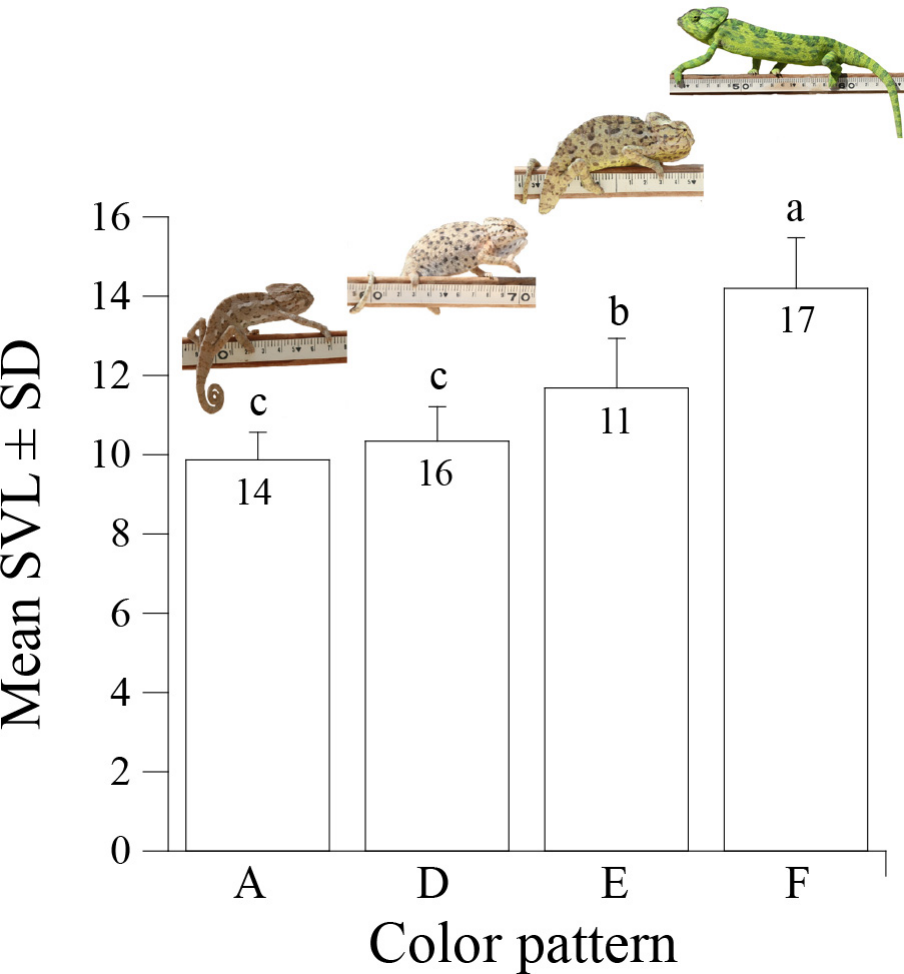
The association between male courtship color patterns and SVL. Uppercase letters denote males in color pattern A, D, E, and F. Mean SVL values not connected by the same lowercase letter are significantly different (Tukey HSD, P < 0.01).

During the breeding season most males temporarily altered their present color pattern (i.e., green or brown) while courting. The temporarily color change was not a result of darkening but of a change in body color pattern. We identified three distinctive temporary courtship color patterns, which were associated with individual's seasonal body color (i.e., green or brown) and, with body length (Fig 3). Small brown males used courtship patterns D, medium size brown males used courtship pattern E, and large green males used courtship pattern F (Fig 1, Fig 3 and S1 Fig). Snout-vent length (SVL) significantly differed among all courtship color patterns (F_3,54_ = 55.4, P<0.0001), except between pattern A and D (Tukey HSD, P = 0.62; Fig 3).

The SVM classification rate of color patterns exhibited distinctive color patterns for both color images and grayscale images, which significantly distinguished between each male size group (Table 1). Calculating leave-one-out classification success rate allowed us also to determine whether the short-term color patterns that males display while courting (Patterns D, E and F) were significantly and distinctly different from the brown or green long-term color patterns that males display throughout the breeding season. Classification rate of courtship color patterns for small and medium-size males (patterns D and E, respectively) significantly differed from their color patterns during the breeding season (brown color displayed by patterns A, B and C) for both color and greyscale images (Table 1 and Fig 1). The significant differences in Table 1 were valid even after sequential Bonferroni correction except for the comparison between patterns D and E in grayscale. In large males, which appeared in pattern F, the courtship color pattern was simply a brighter version of their breeding season color pattern and therefore was not classified a priori as a different color pattern (Table 1). Furthermore, classification success between courtship color pattern of small, medium, and large males (patterns D, E and F, respectively) for both color and grayscale images was high (mean classification success for color pattern = 0.94) and significantly larger than that expected by random (Table 1). Courtship color patterns of small and medium-size males (patterns D and E) was dull brown, though the dorsal surface in pattern E was bright yellow. In contrast, courtship color pattern of large males (pattern F) was bright and contrasting (Fig 1). Our findings suggest that these unique breeding season and courtship color patterns are tightly linked with body size classes, and that there is no overlap between the long-term breeding season and short-term courtship color patterns within size classes, except in large males.

**Table 1:** Leave-one-out classification success rate and P-value between relevant pairs of patterns using the Support Vector Machine (SVM) classifier.

	Color images		Grayscale images	
Patterns	Success rate	P	Success rate	P
A—C	0.735	**<0.0001**	0.853	**<0.0001**
A—D	0.681	**<0.0001**	0.787	**<0.0001**
A—F	0.957	**<0.0001**	0.674	**0.003**
B—C	0.630	0.871	0.593	0.796
B—D	0.750	**<0.0001**	0.875	**<0.0001**
B—F	1.000	**<0.0001**	0.615	**0.007**
C—E	0.706	**0.003**	0.588	0.371
C—F	0.818	**0.0022**	0.636	**0.007**
D—E	0.833	**0.0002**	0.633	*0.046
D—F	0.971	**<0.0001**	0.771	**<0.0001**
E—F	0.862	**<0.0001**	0.724	**0.003**

The analyses were performed on the same images in color and grayscale. Pattern pairs that significantly differ are denoted in bold. A nonsignificant P value after sequential Bonferroni correction is denoted by an asterisk.

### Alternative male mating tactics and body color

Towards the breeding season, many of the smaller males (i.e. one-year-old individuals) altered their body color from green to brown (patterns A and B, Figs 1 and 2), a seasonal color patterns that resembled that of females (Table 1). To further test for similarity in appearance between brown males and brown females, we used the SVM to classify brown males, using both color images for color and grayscale images for pattern alone. Again, SVM was used as the underlying classifier. During the classifier training stage, we used two classes: brown females (n = 21 different females) and green males (n = 17). Using the leave-one-out classification rate, these two categories were significantly different both in color and pattern (randomization test; P<0.0001) and in pattern alone (P = 0.003). Next, we introduced images of 47 different brown males (i.e., patterns A and B), which were assigned as unknowns, and allowed the classifier to assign them as male or female. The SVM classifier assigned 94% of the color (i.e., color and pattern) images and 66% of grayscale (i.e., pattern alone) images of the unknowns as female. Moreover, using the leave-one-out test, the unknown group as a whole (i.e., brown males) could not be statistically separated from females based on color (P = 0.217) or grayscale (P = 0.073). These results indicate that small brown males in their first year resemble the appearance of brown females, and largely differ in color and pattern from the larger green males.

Furthermore, large green males displayed mating behavior that was never observed in smaller brown males. During the breeding season, we captured 36 large green males, of which 11 (31%) were observed asleep in the immediate vicinity of a female (<10 cm away). On the following morning, 10 of these 11 males were observed moving on the branches in close proximity to the female. Therefore, we considered this behavior of large green males as mate guarding (following [32]). In contrast, brown males were never observed sleeping near a female (n = 47, χ^2^_1_ = 16.6, P<0.0001).

In a series of male-male encounter trials, we intended to test for an association between body color and mass or SVL. Larger males were heavier (Log-Log transformation, r^2^ = 0.93, F_1,184_ = 2364, P<0.0001). Our trials revealed several behavioral trends associated with body color and mass. 1) Perch height preference—lone heavy green males tended to perch higher than small lighter males (logistic regression, tree height: likelihood ratio χ^2^_1_ = 6.3, P = 0.012, body mass: LR χ^2^_1_ = 11.7, P = 0.0006, n = 38). A similar trend was observed for a pair of males on the same tree; green heavier males preferred to perch higher than brown lighter males (logistic regression, tree height: LR χ^2^_1_ = 8.9, P = 0.003, body mass: LR χ^2^_1_ = 7.0, P = 0.008, n = 23). 2) Tree descent—during male-male encounter trials, one individual often descended the tree to the ground without any agonistic escalation within the first 15 min. Heavier males were the ones that tended to win male-male tournaments and stay on the tree (logistic regression, likelihood ratio χ^2^_1_ = 8.6, P = 0.003, n = 28). Green-body males tended to remain on the tree and brown-body males to descend (Contingency table, χ^2^_1_ = 7.2, P = 0.007, n = 36). Finally, green males won 9 out of 11 direct matches between green and brown males, an outcome that is significantly greater than expected by random (binomial test, P = 0.027). 3) Body inflation—during male-male encounters green-body males inflated their body significantly more often than their brown-body opponents (χ^2^_1_ = 5.4, P = 0.021, n = 36). The variables body inflation and tree descent were uncorrelated (χ^2^_1_ = 0.4, P = 0.551).

### Female mating preference

Out of 42 encounters between a single male and a single female (n = 42 males), females copulated with 5 out of 12 green males and 15 out of 30 brown males; a roughly equal rate for both male color forms (χ^2^_1_ = 0.24, P = 0.625). Thus, females did not show any mating preference for either male body size or male body color class.

## Discussion

### The link between body color and individual size

Our results show that during the breeding season larger males retained their year-round regular green body color, while smaller ones shifted to brown. A seasonal shift in body color as a function of body size has also been observed in other lizard species [12, 16, 18, 26–27]. These seasonal body color changes are referred to as morphological color change [44–45]. Morphological color change in the common chameleon during the breeding season has been reported previously [29, 31], although not in relation to other characters. In this study, we related male body color to body size. In this respect, the body color pattern may enhance the detection of body size from a distance. In some chameleon species, male-male encounters during the breeding season frequently result in antagonistic interactions, which may escalate to intense biting and can result in severe injury [46]. Assessing body size through color from a distance might reduce the chance of an aggressive encounter.

In addition to the seasonal change in body color, chameleons are known for their rapid color change during social interactions as a means of communication [29, 47–50]. Males may display distinctive color patterns during agonistic encounters with other males and while courting females, as was shown in *Anolis* [26], and in several chameleon species [29, 50–51]. Such rapid, temporary changes in body color patterns are termed physiological color change [44–45]. Greenberg [52] suggested that long-term morphological color changes might interfere with the expression of the rapid physiological color pattern change, which is often used for social signaling. Therefore, we hypothesized that the seasonal morphological color patterns we observed in chameleon males might also affect the momentary change in color patterns used during social encounters with females and other males. We found that each distinctive seasonal long-term morphological color pattern elicited a distinctive short-term physiological color pattern during courtship and male-male encounters, and that both short and long-term color patterns were dependent on body size. In addition, courtship and male-male encounter color patterns of small and medium size males were found to be duller than those of larger males, which were much brighter. Observations in other chameleon species such as *Chamaeleo hoehnelii* [53], *Chamaeleo jacksonii* [54], *Chamaeleo calyptratus* [46], and others [50] have shown that the dominant males exhibit brighter and more distinctive color patterns during courtship and male-male encounters than do non-dominant males. Stuart-Fox and Whiting [48] reported that encounters between male chameleon opponents could escalate in many cases to biting, which can result in severe injury. We suggest that the ability to assess visually a conspecific based on body color, ornamentation, and brightness, before and during an encounter, might enhance body-size distinctiveness and aid in avoiding escalation and possible injury [47].

### Female-like appearance of males

The possible role of female-like appearance by sneaker males is to enable them to move about freely and gain access to females in the range of resident males, while on the other hand keeping away and avoiding direct contact or any other physical interaction with the dominant males. Such tactics can be successful if female-like appearance is only at the visual level and not at the olfactory level [12]. Our results show that during the breeding season first-year small brown-body males resembled the color pattern of brown-body females. Support for the success of such tactics also comes from two observations of large green males attempting to copulate with small brown males. These she-males have nonetheless also been observed attempting to avoid the presence of dominant males. The resemblance of small brown-body males to females has been documented in several other lizard species [12, 26–27], and is considered a tactic that could reduce the chance of a small male being physically attacked by a dominant male during courtship attempts [12, 15]. In some species, males might alternate between dominant and female-like tactics according to fluctuations in competitive status among the males and mating opportunities [55–56]. However, we did not observe shifts in males between dominant and female-like displays either during the trials or during the breeding season. Large green males remained significantly different in color pattern from small brown-body males and females.

The proportion of brown females in our study population during the breeding season was 37%. Hypothetically, small males could have stayed either green or brown and still resembled some of the females in the population. The only advantage we see in being brown in color is that of the distinctiveness from the green pattern of dominant males. The brown color of small males, which may not be considered as a threat, might thus reduce even further their chances of being attacked by a dominant male. However, our results show that both smaller males and females tend to become brown during the breeding season. This result implies that small males do not intentionally mimic the appearance of females but change to the brown morph, which is the common signal in this population for subordinate status, probably to minimize conflicts with larger males.

### Male color pattern, social status, and alternative mating tactics

Cuadrado [32, 57] showed that larger chameleon males employed a distinctive mating tactic in which they guarded females, as in some other lizard species [58–59]. Our observations also suggest that only large males, which are green throughout the breeding season, guarded females. Cuadrado [32] also reported that while smaller males did not guard females, they did approach receptive females following removal of the guarding male. These observations suggest that smaller brown-body males exercise no guarding behavior but may behave as "sneaker" breeders, as has been documented for other lizard species [7, 22]. Our findings, combined with those reported by Cuadrado [32, 57], suggest that the alternative mating tactics demonstrated by common chameleon males are expressed in long-term morphological change in body color and determined by body size.

Further, our findings show that during the observed male-male trials, males of different body color and body size classes behaved in different ways. Larger green males perched higher than smaller brown males, did not descend the tree during trials, and inflated their body significantly more frequently than the smaller brown males. These dominant behavioral patterns displayed by larger green males during male-male trials have been observed in other lizards as well. Studies on the arboreal lizard, *Anolis carolinensis*, showed that green dominant males remained on the higher branches, while brown subordinates were excluded from these perches and driven off the tree by the dominant male [59–60]. Body-inflation too is known as a dominant display in several chameleon species (*C*. *chamaeleon* [29, 32]; *Chamaeleo calyptratus* [47]; *Bradypodion spp*: [48]). Our current findings suggest that males of different body color and body size behave differently in male-male encounters, and their behavioral repertoire indicates that color and size classes reflect social status, in which the larger green males are the dominants and the smaller brown males are the subordinates. Taken together, mating tactics in this system seem to be largely determined by physical size and social rank.

### Female mate choice

Sexual selection theory predicts that females should prefer to mate with the larger (i.e. dominant), stronger (i.e. territorial), and older (i.e. higher survivorship) males. However, many studies have concluded that female lizards generally do not exhibit mate choice, and that sexual dimorphism in males is generally attributable to male–male competition [60–63]. Nonetheless, a few studies have found evidence of female mate preference in natural populations of lizards, primarily based on size of the males [23, 64–67]. A recent study showed that green lizard females preferred males with higher UV reflectance [68]. There is, however, no experimental evidence for female preference among different male color morphs [27, 69]. In the present study, females showed no mating preference for particular males, either in relation to different mating tactics or in relation to different courtship color patterns. Our findings thus do not support the hypothesis that females would prefer to mate with the higher social status class, the green dominant males. However, females may copulate indiscriminately to store sperm from multiple males and increase genetic diversity of their clutch [70]. On the other hand, temporary courtship color patterns, which are also displayed during male-male encounters [29, 71], might play a role in male-male social signaling and competition for access to females [72].

### Synthesis

Establishing a link between color pattern and mating strategies is dependent on grouping individuals into color pattern classes (Fig 1). Spectrophotometry has been extensively used for study body color of animals, including colors patches in chameleons [45, 49–50]. However, spectrophotometry is only suitable for measuring point samples and not complete body color patterns [73], especially when patterns are composed of a complex mix of colors. Spectrophotometry is also limited to subjects who are restrained or inert during measurements and unsuitable for animals that can rapidly change the color and patterns in response to the presence of the researcher [73]. These limitations of spectrophotometry make this method inappropriate to the current study. *Chamaeleo chamaeleon* individuals have complex color patterns and tend to change body color rapidly when approached or handled. Therefore, in this study we used a combination of photography and computer vision techniques as an alternative approach, which has been successfully employed before [37, 38, 73], also for studying color patterns of chameleons [46, 74]. This combined approach worked well in our study because it supported our manual classification except for one case (patterns B and C). Documenting color patterns of individuals using a camera from a distance was essential for our study in order to completely eliminate the influence of the observer on color change. Furthermore, our methodology allows to analyze the complete pattern on the body and not only specific uniform patches, and enabled statistical unbiased classification of both color and pattern of individuals into color pattern classes. Such approach is appropriate for the analysis of complex or irregular color patterns in animals.

Our results support the hypothesis that the shifts in male body color are associated with social status and represent alternative mating tactics. We have shown that, as predicted, during the breeding season the larger males are the dominants and the smaller males are the subordinates. These differences in body sizes are associated with color patterns, such that smaller and medium-size males display brown color patterns while larger males are green. Thus, during the breeding season size and color of males seem to be correlated. In addition, smaller males seem to be similar in appearance to small females. Further, we have shown that temporary courtship color patterns also correspond to body size differences. These results demonstrate a coupling between long-term seasonal body colors (i.e., green vs. brown) and temporary color patterns displayed while courting and in male-male interactions. Why should such signal coupling exist in chameleons? Below we provide two, alternative, explanations.

1. Studies on vocal communication have shown that signal detection theory often applies in noise environments [75–76]. Many birds, for example, repeatedly sing the same song in order to ensure detectability by the receiver. Unlike most animals, the color and pattern shifting body of chameleons is like an electronic billboard that alternates between advertisements. With patterns and colors on the chameleon body able to change momentarily, transitional stages could present a mix of patterns, a noisy signal that is difficult to interpret. The coupling between long-term body color and short-term courting pattern could be viewed as signal enhancement, which is often expressed in repetition or redundancy [77]. Thus, chameleons reinforce their signal of social status when it is most important, during social encounters.

2. Alternatively, we could view the coupling of the long and short-term signals as a two-stage communication system, with each stage transmitting different information: first, the long-term color discriminates between dominant and non-dominant chameleons; and second, the short-term pattern that clearly defines the sex (i.e. only males have the courtship display) and size range (i.e. each size class animals employs a distinctive courtship display) of the signaler. Such a two-stage communication system might define the quality of the partners, increase body-size distinctiveness, and aid in avoiding escalation and possible injury among males.

Our current data do not permit a clear distinction regarding which of the above explanations better fits our observations. Furthermore, we have extrapolated from the findings of Cuadrado's [29–33] studies in Spain on *Chamaeleo chamaeleon* to the sneaking and mate-guarding behavior of the same species in Israel. A more elaborate set of behavioral trials is thus required in the future in order to better understand the function of this signal coupling in our chameleon population.

## Supporting Information

S1 Fig Differences in male courtship color patterns. (PDF)

S2 Fig The white ruler, used in this study as a standard, calibrated to the white (bottom left cell) of the GretagMacBeth ColorChecker chart. (JPG)

S3 Fig Recaptured male that had shifted between long-term body color morphs. (PDF)

S1 Table Excel sheets that contain all the data used in our analyses. (ZIP)
