# Supplementary material for: Alternative Mating Tactics in Male Chameleons (Chamaeleo chamaeleon) Are Evident in Both Long-Term Body Color and Short-Term Courtship Pattern
Source: PLoS One. 2016 Jul 13;11(7):e0159032. doi: 10.1371/journal.pone.0159032 (PMC4943735; doi:10.1371/journal.pone.0159032)
Supplement: S1 Fig — (PDF) [file pone.0159032.s001.pdf]

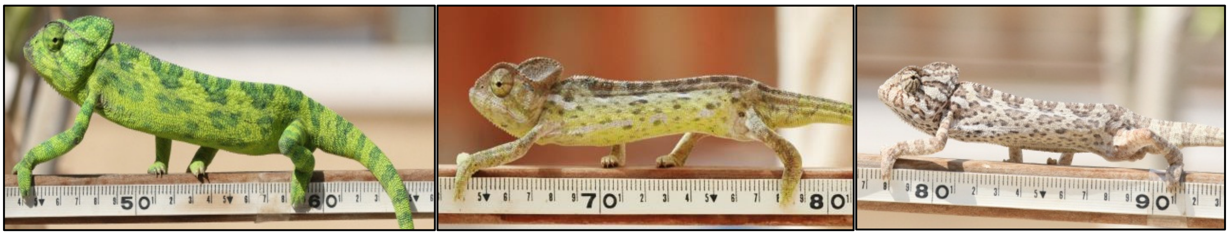

Fig. S1: Differences in male courtship color patterns. On the left, large dominant green male in pattern F, in the middle, a medium size brown male in pattern E, and on the right, a small brown male in pattern D.
