# Supplementary figures and images for: Alternative Mating Tactics in Male Chameleons (Chamaeleo chamaeleon) Are Evident in Both Long-Term Body Color and Short-Term Courtship Pattern
Source: PLoS One. 2016 Jul 13;11(7):e0159032. doi: 10.1371/journal.pone.0159032 (PMC4943735; doi:10.1371/journal.pone.0159032)

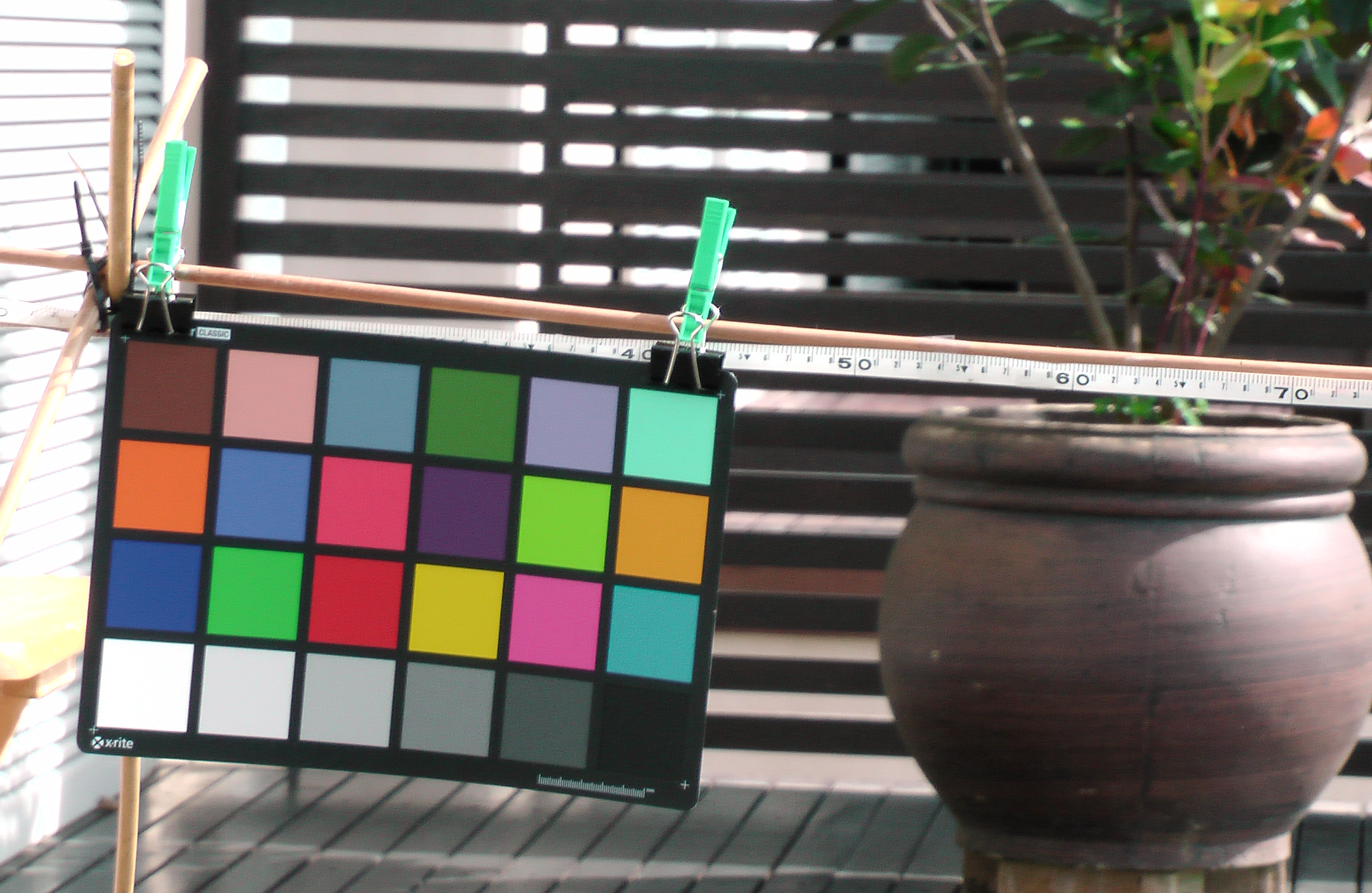

Supplement: S2 Fig — (JPG) [file pone.0159032.s002.JPG]
