# Supplementary material for: Alternative Mating Tactics in Male Chameleons (Chamaeleo chamaeleon) Are Evident in Both Long-Term Body Color and Short-Term Courtship Pattern
Source: PLoS One. 2016 Jul 13;11(7):e0159032. doi: 10.1371/journal.pone.0159032 (PMC4943735; doi:10.1371/journal.pone.0159032)
Supplement: S3 Fig — (PDF) [file pone.0159032.s003.pdf]

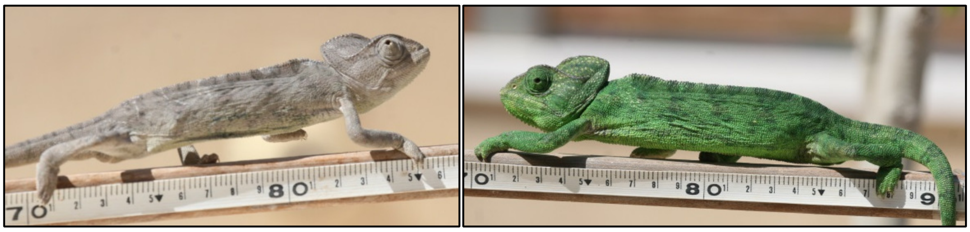

Fig. S3: Recaptured male that had shifted between body color morphs. On the left, the male is in a brown morph during a second or third breeding season, and on the right the same male in a green morph during the following breeding season.
